# Hospitals, Charity, and Organisations

**Published:** 1897-09-25

**Authors:** 


Vol. XXII.—No. 574.] [Sept. 25, 1897.

### Hospitals, Charity, and Organisations.

The possible influence, sometimes for good, sometimes for evil, of the intrusion of great organisations between hospitals and their supporters is a topic which requires to be looked at from various points of view.

On the one hand it has to be remembered that there is an immense fund of charitable feeling in the country, coupled with an immense, an immeasurable, amount of ignorance as to the best way of doing good, and that this fund is tapped to an extent that could not be accomplished in any other way by the establishment of organisations, guaranteed by responsible names, and undertaking to distribute to the best of their ability any funds placed in their hands. Again, he would be blind who would maintain that our hospitals are perfect and without fault, and it is clear that by the exercise of careful judgment in the distribution of such funds an influence for good may be constantly brought to bear upon their administration. These are reasons which may properly be urged in favour of the intervention of great collecting organisations between the subscribers and the hospitals. They are reasons which we have again and again urged in support of the Hospital Sunday Fund, and we have urged them without fear, because we have faith in its management. But we cannot shut our eyes to the fact that such organisations as interfere with the freedom of charity, although powerful for good so long as they are well managed, are pregnant with infinite possibilities of mischief should they fall into wrong hands. The matter, however, is still more serious in regard to such associations as have their root among those classes for whose benefit the hospitals ostensibly exist, and flourish by their exploitation of the "ticket" or "letter" system—that evil parasite by which the charitable character of so many of our medical institutions tends to be destroyed.

In origin and in intention the ticket system was good. The very form still in use in many cases shows that its whole aim was to ensure that the giver of the tickets should investigate each case, and should, from his personal and local knowledge, restrict their use to those who were truly "objects of charity" or "persons deserving of relief."

All this, however, is now a thing of the past. Even with the old-fashioned subscriber, the filling up of the ticket has too often become a mere matter of form, and in the sale of tickets to associations for the use of their members all pretence of charity is dropped—it is a simple bargain, so many pounds, so many tickets.

Perhaps the most striking example of the influence which may be obtained by a working man's association in the control of hospital affairs is to be found in Birmingham, where, by dint of an annual donation of £10,000—a noble sum, be it said—from the Hospital Saturday Fund, one man is able to lead by the nose all the hospital managers of the town. It is a grand position, and certainly we should be the last to grudge or to belittle the honour due to Mr. Smedley for bringing this fund to its present state.

The fund, however, as it stands, is a dangerous tool, which, if misapplied, might do infinite harm to the medical charities of the town. Mr. Smedley not only has faith in the working classes, but has great influence over them; he has a clear head, and we must hope he will steer a clear course. But what might happen in other hands must be a matter of the gravest doubt. One cannot avoid the thought that even he is quite as much in love with the organisation of the Fund as with the hospitals for which it was founded, and there seems but little to prevent its whole force being diverted towards the development of a great co-operative medical association if he should choose to turn the rudder in that direction. It must always be remembered that the money given to the Saturday Fund is a surplus, the first charge on the shop subscriptions being the money required for the purchase of such hospital tickets as are wanted by the subscribing members. Again, of recent years the free gift to the hospitals has not been the whole Hospital Saturday Fund, but a surplus after payment has been made for the support of the convalescent homes run by the fund itself. So far as about half the money collected in the workshops is concerned, it is devoted entirely to co-operation purposes, which come as a first charge upon it. These include the support of convalescent homes belonging to the fund, a payment to the District Nursing Society for the services of nurses to the subscribers to the fund, and a sum of about £6,000 which, without going into the fund at all, is used for the purchase of hospital tickets. Now it must be borne in mind that this free sale of tickets tends to entirely undermine the charitable character of the hospitals, and reduce them to mere provident institutions of the worst form, *i.e.*, institutions without a wage limit. Hospital managers and medical staffs no doubt protest. But they may kick as they like. What can they do against an annual tip of £10,000? This is what it really is. The Birmingham medical institutions are tied to the Saturday Fund by this free gift, and for the sake of it they are forced to watch the slow absorption of their charities by a great co-operative movement. This is what a working man's organisation does at its best. What it will do at its worst we do not know.

